# Performance of Preschoolers’ Mothers and Senior Dental Students After Receiving Training on Fluoride Varnish Administration

**Published:** 2017-07

**Authors:** Simin Zahra Mohebbi, Samaneh Razeghi, Zahra Chinipardaz, Hamideh Soleimannejad, Mohammad Javad Kharazifard

**Affiliations:** 1 Associate Professor, Research Center for Caries Prevention, Dental Research Institute, Tehran University of Medical Sciences, Tehran, Iran; Department of Community Oral Health, School of Dentistry, Tehran University of Medical Sciences, Tehran, Iran; 2 Assistant Professor, Research Center for Caries Prevention, Dental Research Institute, Tehran University of Medical Sciences, Tehran, Iran; Department of Community Oral Health, School of Dentistry, Tehran University of Medical Sciences, Tehran, Iran; 3 DSc Candidate, Department of Periodontics, School of Dental Medicine, University of Pennsylvania, Pennsylvania, USA; 4 Dentist, Research Center for Caries Prevention, Dental Research Institute, Tehran University of Medical Sciences, Tehran, Iran; 5 Epidemiologist, Dental Research Center, Dentistry Research Institute, Tehran University of Medical Sciences, Tehran, Iran

**Keywords:** Fluorides, Child, Preschool, Mothers, Oral Health

## Abstract

**Objectives:**

Fluoride varnish application is an effective way to prevent caries in children. We aimed to educate preschool children’s mothers on how to apply fluoride varnish and compare their performance with dental students and to assess their self-reported competency six months later.

**Materials and Methods:**

Eighty-eight 4–6-year-old children presenting to toy houses of six randomly selected health centers in a non-affluent district of Tehran in 2014 were divided into two groups to receive fluoride varnish by their mothers and students. Mothers and senior dental students participated in a session consisting of lecture, discussion, and demonstration of fluoride varnish application for a child. Then, in three centers, mothers and in others, students applied fluoride varnish for preschoolers and their performance was evaluated. Six months later, mothers were asked to apply fluoride varnish again. Mann-Whitney U test, t-test, one-way ANOVA and logistic regression model were used for statistical analyses.

**Results:**

The mean performance score was 9.74±0.22 (out of 10) for mothers and 9.71±0.15 for students (P=0.89). After six months, the mean performance score was 9.58 for mothers, which was not significantly different from that in the first session. The age and educational level of mothers were conversely correlated to their performance (P<0.05). Of mothers, 96% believed that they were competent to repeat fluoride varnish application for their children.

**Conclusions:**

Mothers’ high performance score and the point that the majority of them felt competent to apply varnish for their children casts light on their potential key role in oral health promotion.

## INTRODUCTION

Early childhood caries (ECC), a serious public health dilemma, can begin as early as the teeth begin to erupt. It often progresses fast and can cause great pain and discomfort for the child [1,2]. If left untreated, it can destruct the child’s teeth, and have a strong and long-term effect on child’s general health and wellbeing [3,4]. ECC is epidemic in several countries. In Iran, the prevalence of ECC is 52% in three-year-old children [5] with a higher prevalence in low socioeconomic groups, who are unable to receive dental care due to substantial costs of treatment [3]. Effectiveness, ease of use and relative safety of fluoride varnish makes it an attractive option compared to other topical fluoride products, such as gels and mouth rinses [6–10]. There is strong evidence that application of fluoride varnish two times a year reduces decayed, missing and filled tooth surfaces by 46% in children and adolescents after a one-year period [11]. Primary preventive dental services are successfully delivered by non-dental professionals [12–14]. A study in Finland revealed the prospering role of parents in administration of topical xylitol to their infants at home leading to considerable improvement in infants’ dental health [15]. Okunseri et al, [16] demonstrated that children’s access to fluoride varnish treatment significantly increased by allowing medical care providers to provide children with fluoride varnish treatment. Some studies in Iran have exhibited that physicians and nurses have positive attitudes and notable willingness to receive training on oral health care since they lack knowledge in this field [17,18]. Mothers play a critical role in providing children with the information and encouragement needed for a healthy lifestyle [19]. In order to provide adequate access to primary caries prevention programs in areas with lower socio-economic status, we decided to educate preschool children’s mothers on how to apply fluoride varnish and compare their performance with dental students. In addition, we assessed mothers’ long-term performance, self-reported competency and satisfaction with the course six months later.

## MATERIALS AND METHODS

### Ethics:

Ethical approval was granted by the Ethics Committee of School of Dentistry, Tehran University of Medical Sciences (Doctoral Thesis No. 5063). The survey was voluntary and the responses were anonymous. All the participants were informed about the objectives and steps of the study.

### Sampling:

The sample of this interventional study was comprised of two groups of mothers and students. A pilot study was performed on 15 students to determine the sample size. To reach a minimum power of 80% (α= 0.05, β= 0.2, difference between means of 24 and standard deviation of 20), the sample size was estimated to be 15 people in each group. For better consideration and loss to follow-up compensation, participants increased to 40. Mothers and their 4–6-year-old children (n=43) presenting to the toys houses of three municipality health centers in a southern district of Tehran (non-affluent area) in 2014 were included in mothers group. Children in three other toys houses (n=45) in the same city district received fluoride varnish by senior dental students of Tehran University of Medical Sciences.

### Study methodology and educational intervention:

Mothers were asked to fill out a self-administrated questionnaire on oral health knowledge, attitude, behavior and demographics before the intervention. Two topics were taught to the mothers in one hour by a faculty member from the Department of Community Oral Health. First, a topic regarding ECC was lectured and discussed using questions and answers. Then, the properties, advantages and method of application of fluoride varnish were presented by a Power Point presentation. After that, for small groups of mothers (n=5), a pediatric dentist demonstrated fluoride varnish application on a child and then mothers were asked to apply varnish for their own children. Fluoride varnish application was evaluated using a “direct observation of procedural skills” (DOPS) checklist by two faculty members. The same process was undertaken for the students group. After six months, in a second session, mothers were asked to review a short instruction relating to fluoride varnish application technique. Then, they applied fluoride varnish for their child and their performance was evaluated using the same DOPS checklist.

### The DOPS checklist and the questionnaire:

To assess the operators’ performance with regard to varnish application, an 11-item DOPS checklist was developed according to the best available evidence [20]. Two faculty members were calibrated during a session to discuss the details of scoring by rating 10 students (actual agreement= 90%). They evaluated the participants’ performance with regard to fluoride varnish application using the DOPS checklist including eleven items. These items were as follows: 1) choose the appropriate equipment meaning gloves, single dose varnish and gauze; 2) communication with the child to achieve his/her cooperation; 3) properly position the child in order to have good access to the child’s mouth; 4) open the fluoride varnish package; 5) blending the fluoride varnish to reach a uniform mixture; 6) clean and remove the plaque on teeth; 7) dry teeth; 8) carry the right amount of fluoride varnish for each quadrant (not too much and not too little); 9) apply a thin layer of the varnish to all surfaces of the teeth; 10) working by quadrant and 11) proper isolation during the work.

In addition to the checklist, a self-administered standard questionnaire was used to assess the demographics of mothers [21] including age and education level of mothers, age and gender of the child and information on knowledge and attitude of mothers towards their children’s oral health, oral health behavior (OHB) of mothers and their children. The knowledge section consisted of seven questions with multiple-choice, or ‘yes’, ‘no’ and ‘I don’t know’ answers. In this section, the questionnaire targeted their knowledge regarding the following: “When is the eruption time of first primary teeth”; “Cariogenic bacteria are usually transmitted from mothers to children”; “Fluoride tooth-paste should not be used for children under 3 years of age”; “The first sign of caries is white spot lesions on tooth surfaces”; “Brushing children’s teeth should begin from the age of 2–3 years old when primary dentition is completed”; “What is the reason of adding fluoride to toothpaste”; and “Usage of fluoride varnish in children under 5 years old can lead to dental fluorosis”. The section on attitude contained two questions: “Children’s oral health problems can lead to general health problems” and “Mothers’ information about their children’s oral health is sufficient”. The provided answers were based on the Likert scale from ‘strongly agree’ to ‘strongly disagree’ including ‘I don’t know’. There were 12 questions about the oral health behavior (OHB): nine questions were on children’s OHB (child’s OHB) and three questions were about mothers’ OHB (mother’s OHB).

The responses for child’s OHB were categorized as follows:

1- Frequency of eating sugary snacks: seldom or never, sometimes but not daily, once a day, twice a day, three times a day, four times a day, or five times a day or more;
2- Frequency of tooth brushing: more than once a day, once a day, 2–3 times per week, once a week, seldom or never.
3- Utilizing fluoride toothpaste: usually or always, often, seldom, never.
4- Teeth cleaning device: toothbrush, gauze, washcloth, water, other devices, nothing.
5- Cooperation during cleaning his/her teeth: usually or always, often, seldom, never.
6- Who brushes the child’s teeth? Adults alone, the child with the help of adults, the child with supervision of adults, the child alone, no tooth brushing.
7- Time of starting to brush the child’s teeth: child’s age by month.
8- Child’s first dental visit: child’s age by month.
9- The reason for the first dental visit: Regular dental checkup, dental or gum problems, referred to a dentist, other reasons, I don’t remember.

The first three items were asked about the mothers’ own behavior regarding themselves too with the same response options (mother’s OHB).

### Mothers’ satisfaction with the educational program and their self-reported competency:

Four more questions were included in the evaluation after six months including: “to what extent are you satisfied with the education received in the first session about fluoride varnish application?”, “to what extent are you satisfied with the written instruction relating to fluoride varnish application technique?” and “to what extent are you convenient with the administration of fluoride varnish?” For the above-mentioned questions, the responses were scored from 0 to 10. The last question was “Do you feel competent to apply fluoride varnish for your child every six months after receiving this educational intervention?” with “yes” or “no” response.

### Statistical analysis:

Responses to knowledge questions were scored as follows: ‘1’ to false, “2” to “I do not know” and 3 to correct answers.

The scores were calculated on a scale from 7 to 21. Next, the total knowledge score was calculated for each participant. We coded the responses to the OHB questions so that higher scores represented more desirable habits, and then calculated two sum variables for mothers’ own OHB (mother’s OHB) and their children’s OHB (child’s OHB) by summing up the scores for questions of mother’s OHB (possible range from 0 to 14) and child’s OHB (possible range from 0 to 40).

The maximum and minimum possible scores for each item in the DOPS checklist were 10 and 0, respectively. Higher scores indicated greater competency in performing the procedure.

The obtained data were entered into SPSS version 18 (Chicago, IL, USA). Linear regression model (backward method) served for multivariate assessment to control for the effects of confounders by a stepwise method applying automatic selection of independent variables. T-test and ANOVA were used to evaluate the differences between the groups. Mann-Whitney test was used to compare performance between the two groups. P-values less than 0.05 were considered statistically significant.

## RESULTS

At baseline, 88 (mothers group=43, students group=45) participants were enrolled in the study; of whom 27 mothers and 43 students attended the evaluation session after six months.

### Mothers’ demographics:

In the mothers group, there were 15 male (34.9%) and 28 female (65.1%) children, whilst in the students group, there were 27 (60%) males and 18 (40%) females. Almost half the children were from families with two children. Except for one child’s father in the mothers group who was illiterate, all parents were literate. The mean knowledge score of mothers was 16.1±2.06 out of 21. In the attitude section, most of the mothers believed that dental and general health are related (90.5%). Only 40.5% of mothers believed that their information about their children’s oral health was sufficient. The mean score of OHB was 18.9±5.73 (out of 31) for the children and 8.1±1.63 (out of 14) for the mothers. The demographics of the mothers’ group are shown in Table 1.

**Table 1.** Demographics of families and oral health behavior of mothers attending the intervention (mothers group, n=43)

**Demographics of families and oral health behavior of mothers**	**N (%)**
**Level of education of mothers**^†^	
Illiterate	0 (0.0)
Primary School	3 (7.3)
High school and high school diploma	23 (56.1)
Associate degree	3 (7.3)
Bachelor’s degree	12 (29.3)
Master’s degree	0 (0.0)
Doctorate degree	0 (0.0)
**Children’s oral health problems can lead to general health problems**^††^	
Strongly agree	21 (50)
Agree	17 (40.5)
Don’t know	3 (7.1)
Disagree	1 (2.4)
Strongly disagree	0 (0.0)
**Mothers’ knowledge regarding their children’s oral health is sufficient**^††^	
Strongly agree	5 (11.9)
Agree	12 (28.6)
Don’t know	11 (26.2)
Disagree	12 (28.6)
Strongly disagree	2 (4.8)
**Child’s gender**	
**Male**	15 (34.9)
**Female**	28 (65.1)

**Child’s age & oral behavior data**	**Mean ±SD**
Child’s age (years)	5.06±1.19
Mother’s OHB*	8.1±1.63
Child’s OHB	18.9±5.73
Knowledge score	16.1±2.06

**Mean age of mothers ± standard deviation (years)**	34.50±4.67

* OHB= Oral health behavior

† Two of the participants did not answer this question

†† One of the participants did not answer this question

### The finding of educational intervention:

At baseline, the mean score of mothers’ performance with regard to fluoride varnish application was 9.74 (out of 10) as measured by DOPS checklist and 9.71 in the students group (P>0.05, Table 2). In mothers group, “choose appropriate equipment” with the mean score of 10 and “dry teeth” with the mean score of 9.17 received the highest and lowest scores according to the DOPS checklist, respectively.

**Table 2.** Comparison of performance of mothers (n= 43) and students (n= 45) with regard to fluoride varnish application at baseline according to DOPS checklist

**Types of Performance**	**Mothers group**			**Students group**						

	**Maximum**	**Minimum**	**Mean**	**Maximum**	**Minimum**	**Mean**
Choose the appropriate equipment.	10	10	10	10	8	9.84
Communication with the child	10	5	9.72	10	7	9.88
Properly position the child	10	8	9.92	10	8	9.81
Open the fluoride varnish package	10	9	9.95	10	7	9.84
Blending the fluoride varnish to reach a uniform mixture.	10	8	9.95	10	5	9.33
Clean and remove the possible plaque on teeth	10	8	9.80	10	7	9.80
Dry teeth.	10	5	9.17	10	5	9.35
Carry a right amount of fluoride varnish for each quadrant.	10	5	9.52	10	5	9.66
Apply a thin layer of varnish to all surfaces of the teeth.	10	5	9.65	10	8	9.93
Working by quadrant.	10	7	9.77	10	5	9.75
Proper isolation during the work.	10	1	8.42	10	5	8.84

In students group, the highest score related to “apply a thin layer of varnish to all surfaces of the teeth” and the lowest score to “blending the fluoride varnish to obtain a uniform mixture” with the mean score of 9.93 and 9.33, respectively. The linear regression result of controlling for demographic variables revealed that applying fluoride varnish correctly by mothers was conversely associated with age and educational level of mothers (Table 3).

**Table 3.** Linear regression models controlling for demographics* on factors associated with mothers’ performance with regard to fluoride varnish application. Model a) immediately after intervention, Model b) six months after intervention

	**Model a**			**Model b**						

	**Unstandardized coefficients (B)**	**Standardized coefficients (β)**	**P-value**	**Unstandardized coefficients (B)**	**Standardized coefficients (β)**	**P-value**
**Mothers’ age**	−0.331	−0.309	0.048	−0.340	−0.289	0.042
**Mothers’ educational level**	−1.965	−0.458	0.006	−1.825	−0.395	0.010

* Mother’s age, educational level, knowledge, and oral health behavior; and child’s age, gender and oral health behavior

After six months, in the second session, mothers group was asked to apply fluoride varnish on their children’s teeth again. “Choose appropriate equipment” and “apply a thin layer of varnish to all surfaces of the teeth” with a mean score of 10 and “proper isolation” with a mean score of 8.44 received the highest and the lowest scores, respectively (Table 4). The mean score of performance was 9.58, which was not significantly different from the mean score in the first session (9.74). After controlling for demographic variables, the linear regression model revealed that the age and the educational level of the mothers were conversely related to appropriate fluoride varnish application six months after the first instruction (Table 3).

**Table 4.** Assessment of mothers’ performance with regard to fluoride varnish application after six months according to DOPS checklist (n=27)

	**Maximum**	**Minimum**	**Mean**
Choose the appropriate equipment	10	10	10
Communication with the child	10	5	9.62
Properly position the child	10	8	9.77
Open the fluoride varnish package.	10	9	9.96
Blending the fluoride varnish to reach a uniform mixture	10	0	9.03
Clean and remove the possible plaque on teeth	10	5	9.74
Dry teeth	10	5	9.48
Carry a right amount of fluoride varnish for each quadrant	10	8	9.70
Apply a thin layer of the varnish to all surfaces of the teeth	10	10	10
Working by quadrant	10	5	9.70
Proper isolation during the work	10	3	8.44

### Mothers’ assessment of educational intervention and their self-reported competency:

In the context of satisfaction with the education received in the first session about the fluoride varnish application, 74% of mothers voted 10 (out of 10), 11% and 15% voted for 9 and 8, respectively. Concerning the level of satisfaction with the instruction relating to the fluoride varnish application technique in the six-month follow up, 78% of mothers scored 10, 11% scored 9 and the rest of them scored 8. After six months, amongst mothers, 96% believed that they are competent to repeat fluoride varnish application for their child every six months. Regarding the convenience of applying fluoride varnish, more than half of the mothers (55.6%) scored 10 (out of 10), 40.7% scored higher than 7 and only one mother scored 5.

## DISCUSSION

Many studies have been conducted to determine the notable effect of fluoride varnish on prevention of dental caries in children [22–24]. Since management of younger children is more difficult for health professionals and dental staff, we decided to educate mothers of preschool children who are more dedicated and will spend enough time and patience with their child. Our results revealed the mothers to be competent and eager to perform fluoride varnish application. The point that they could repeat the procedure after six months after reviewing a simple written instruction was promising. According to the “Ottawa statement” [25] enabling people to gain the knowledge and skills to improve and maintain their oral health is critical. This can be achieved by providing information, education and skills for oral health promotion. In the current study, the educational intervention took advantage of integration of multiple teaching methods. The main concepts of ECC were taught to participants by traditional lecture method. Although the usefulness of other teaching methods has been widely examined, the traditional lecture method still remains an important way to convey information [26]. In order to make learning environments more interactive and improve long-term retention of information, participants were also asked to discuss the issue more carefully and then we demonstrated the application of fluoride varnish in small groups. After receiving such education, mothers had a good performance in applying fluoride varnish for their preschool children as compared with senior dental students who are qualified to do so. Some studies were performed on evaluating non-dental professionals’ performance to administrate fluoride varnish in order to prevent ECC. For example, Rozier et al, [27] reported that physicians and other primary health care professionals could expand their roles to include a clinical caries preventive procedure, and integrate these preventive dental services into their practices. This program substantially increased access to preventive dental services for young children whose access to dentists was limited. The results from a randomized controlled trial revealed that a relatively high proportion of medical practices were capable of adopting preventive dental services [22]. After six months, mothers’ performance was good. Although not significant, they even got higher scores than dental students (9.74 vs 9.71) in their performance which might be due to the dedication and patience for one’s own child. Thus, mothers as facilitators to administrate fluoride vanish can help prevent ECC. This result is parallel with the study conducted by Mäkinen et al, [15] who showed considerable improvement in dental health of infants by application of at-home topical xylitol by parents. We targeted a sample from health centers in a southern Tehran district for this evaluation because low-income families are prone to high rates of ECC. Wamala et al, [28] explained the reason for poor oral health among socioeconomically deprived people to be insufficient access to dental care services. Their study highlighted the need for an urgent public health intervention which might increase access to dental care and reduce inequalities in oral health. Thomson et al, [29] in a cohort study concluded that children with low socioeconomic status were more likely to lose a tooth due to caries, and had a higher prevalence of periodontitis in adulthood. Our research was focused on assigning more responsibilities to parents of preschool children with low socioeconomic status to promote oral health in the community. We chose fluoride varnish as a preventive treatment since the efficacy and safety of fluoride varnish have been well proven [30, 31]. Saied-Moallemi et al, [19] demonstrated that mothers’ positive attitude towards oral health had a significant influence on their children’s oral health behavior. They suggested considering and advocating the noticeable potential of mothers for developing oral health promotion programs for children and adolescents. In the present study, mothers were educated about ECC by lecture and presenting pictures from early to advanced stages of ECC. However, mothers’ knowledge and attitude toward oral health were moderate. This could be due to the prevalence of ECC in preschool children and high cost of dental treatment which might have obliged mothers to seek information in this regard. Only age and educational level of mothers were conversely related to correct application of fluoride varnish in two sessions, which means that younger mothers with lower level of education better applied fluoride varnish. We may conclude that these mothers had greater incentives for applying fluoride for their children at no cost. After six months, most mothers (96% of participants) claimed that they were competent to apply fluoride varnish for their children every six months.

This study had some limitations. Some mothers did not attend the second session due to the lack of cooperation of authorities in one of the toys houses. However, at first, we intentionally increased the sample size to compensate for the possible loss to follow up. Another limitation was no response to some questions of the self-administrated questionnaire and over and under-reporting in a few cases. Finally, our research was conducted only in Tehran; therefore, it is possible that our findings may not be generalizable to other provinces. However, Tehran is one of the biggest cities and the capital of Iran with ethnically diverse population; thus, we believe that our results could be generalized to other provinces.

## CONCLUSION

The present study confirmed the ability of mothers to apply fluoride varnish for preschool children and clarified the point that mothers can have a key role in promoting oral health of their children. Hence, by involving mothers in preventive programs such as fluoride varnish application, we will increase the frequency of fluoride varnish therapy in the community especially in underserved areas.
